# Isolation of Saint Louis Encephalitis Virus from a Horse with Neurological Disease in Brazil

**DOI:** 10.1371/journal.pntd.0002537

**Published:** 2013-11-21

**Authors:** Roberta Rosa, Erica Azevedo Costa, Rafael Elias Marques, Taismara Simas Oliveira, Ronaldo Furtini, Maria Rosa Quaresma Bomfim, Mauro Martins Teixeira, Tatiane Alves Paixão, Renato Lima Santos

**Affiliations:** 1 Departamento de Clínica e Cirurgia Veterinárias, Escola de Veterinária, Universidade Federal de Minas Gerais, Belo Horizonte, Minas Gerais, Brazil; 2 Departamento de Bioquímica e Imunologia, Instituto de Ciências Biológicas, Universidade Federal de Minas Gerais, Belo Horizonte, Minas Gerais, Brazil; 3 Laboratório de Saúde Animal, Instituto Mineiro de Agropecuária, Belo Horizonte, Minas Gerais, Brazil; 4 Departamento de Biologia Parasitária, Centro Universitário do Maranhão, São Luis, Maranhao, Brazil; 5 Departamento de Patologia Geral, Instituto de Ciências Biológicas, Universidade Federal de Minas Gerais, Belo Horizonte, Minas Gerais, Brazil; University of Texas Medical Branch, United States of America

## Abstract

St. Louis encephalitis virus (SLEV) is a causative agent of encephalitis in humans in the Western hemisphere. SLEV is a positive-sense RNA virus that belongs to the *Flavivirus* genus, which includes West Nile encephalitis virus, Japanese encephalitis virus, Dengue virus and other medically important viruses. Recently, we isolated a SLEV strain from the brain of a horse with neurological signs in the countryside of Minas Gerais, Brazil. The SLEV isolation was confirmed by reverse-transcription RT-PCR and sequencing of the E protein gene. Virus identity was also confirmed by indirect immunofluorescence using commercial antibodies against SLEV. To characterize this newly isolated strain *in vivo*, serial passages in newborn mice were performed and led to hemorrhagic manifestations associated with recruitment of inflammatory cells into the central nervous system of newborns. In summary this is the first isolation of SLEV from a horse with neurological signs in Brazil.

## Author Summary

St. Louis encephalitis virus (SLEV), a member of the *Flavivirus* genus, which includes West Nile encephalitis virus, Japanese encephalitis virus, Dengue virus, and other medically important viruses, is a cause of encephalitis in humans and animals. SLEV is considered endemic in the Americas, and currently there is no vaccine or specific treatment available for controlling of preventing SLEV-induced encephalitis. In this study we describe the first isolation of SLEV from an adult male horse with neurologic disease, which was further characterized by molecular and serological methods. Phylogenetic analysis of a 903 base pairs amplified sequence from partial Envelope (E) gene region indicated that the isolate from the horse was within the cluster of the VB genotype. In addition, inoculation of the SLEV isolate intracranially in newborn mice resulted in circulatory and neurological changes. This is the first report of isolation of SLEV from a horse with neurological disease in Brazil.

## Introduction

St. Louis encephalitis virus (SLEV) is a mosquito-borne virus that causes human and animal encephalitis in the Western hemisphere. SLEV is considered endemic in the Americas, with encephalitis cases being diagnosed from Canada to Argentina [1]–[3]. There is no vaccine or treatment available for St. Louis encephalitis.

SLEV is a single-stranded positive sense RNA virus, with approximately 50 nm in diameter and a genome of 11 kb. SLEV is a member of the *Flavivirus* genus in the *Flaviviridae* family, together with several important pathogens such as West Nile virus (WNV), Japanese encephalitis virus (JEV), Dengue virus (DENV), Yellow fever virus (YFV) and others [4], [5]. Viral life cycle is enzootic and birds are the natural amplifying host [6]. Other vertebrates (e.g. wild animals, horses, and humans) are considered accidental/final hosts [7]–[9].

Human infections with SLEV are mostly asymptomatic. Infected individuals can present mild malaise or flu-like symptoms, especially young or middle-aged patients [6], [10]. Severe cases are clinically characterized by high fever, neurological dysfunction, altered consciousness, and headache; which are accompanied by encephalitis or meningoencephalitis that affects more often the elderly [11]–[13]. Lethality rates in severe cases can reach 30%, and are associated to direct damage to the central nervous system (CNS) [3]. Acute illness can be followed by prolonged convalescence with cognitive and psychosocial deficits for over a year [6], [14]. Disease in wild or domestic animals has not been described, although many species are infected or are serologically positive for SLEV in endemic areas [6], [15]–[19].

SLEV has been detected in Brazil for over 40 years, isolated from arthropods [19] or by serological surveys in birds [20] and mammals [18], [21]. SLEV was isolated from two patients in the Amazon region in 1970's [22], [23] and isolated again from a dengue-suspected patient in Southeastern Brazil, in the early 2000's [2]. Interestingly, SLEV infections in humans were identified in southeast Brazil in the following years, under an outbreak of DENV-3, together with the first a human case of DENV-3 and SLEV co-infection [24], [25].

Here we describe the first isolation of SLEV from a horse with neurological signs in Brazil. SLEV identity was confirmed by molecular and serological techniques, and by inoculation of newborn mice. Our findings highlight the importance of effective arboviral surveillance.

## Materials and Methods

### Ethics statement

Our animal study followed national guidelines (Law number 11.794, 8/10/2008), which governs the use of animals for experimental procedures. All experimental procedures were approved and complied with the University of Minas Gerais (UFMG) Committee for Ethics in Animal Experimentation (CETEA) regulations, under protocol number 163/2011.

### Mice

Pregnant female mice were acquired from Centro de Bioterismo (CEBIO) of UFMG (Belo Horizonte, Brazil). Newborn Swiss mice (24 hours old) were used in animal model development experiments. All mice were kept under controlled temperature (23°C) with a strict 12 h light/dark cycle, food and water available *ad libitum*, under specific pathogen-free conditions, in the animal warren at the Departamento de Clínica e Cirurgia Veterinárias of UFMG.

### Sample collection and PCR screening

Brain tissue from horses that presented neurological symptoms before death were sent to Laboratório de Saúde Animal at Instituto Mineiro de Agropecuária (LSA/IMA) in Belo Horizonte, Brazil. Samples that were PCR negative for Rabies virus were sent to Laboratório de Patologia Molecular at UFMG and stored at −80°C. Tissue samples were processed for RNA extraction and screened by nested RT-PCR. There were no tissue samples available for histopathology. Reaction parameters and primers were used as described by Ré and colleagues [26]. Primers used for the initial amplification were SLE 1497 (+) RRYATGGGYGAGTATGGRACAG, SLE 2517 (−) CTCCTCCACAYTTYARTTCACG, and primers for the final amplification were SLE (+) 2002 TGGAYTGGACRCCGGTTGGAAG and SLE (−) 2257 CCAATRGATCCRAARTCCCACG.

### Sequencing and analysis

SLEV RT-PCR amplicon band was purified from an agarose gel and sequenced in Megabace 1000 sequencer. Edited sequences were aligned by CLUSTAL/W, using the BioEdit program, version 5.09. The resulting sequence was deposited in GenBank (accession number KF718857).

A phylogenetic tree was generated using the Molecular Evolutionary Genetics Analysis software (MEGA - www.megasoftware.net), version 4 (MEGA 4) [27]. The neighbor-joining method was used to generate bootstrap of 1,000 replications using p-distance. Nucleotide sequences were used to perform a similarity search in sequence databases, using BLAST algorithm (http://blast.ncbi.nlm.nih.gov/). Construction of the phylogenetic was based on 39 other SLEV sequences available at GenBank, which were distributed within the following genotypes: IA, IB, IIA, IIB, IIC, IID, IIE, IIF, IIG, III, IV, VA, VB, VI, VII, VIIIa; VIIIB and 3 out-groups: WNV, JEV, and DENV, as detailed in Table 1. The isolated viral strain was categorized within genotypes as previously described [19], [28]–[31].

**Table 1:** Viral strains included in phylogenetic analysis.

Genotype	GenBank Accession	Designation*	Reference
I A	AF205455	CA-53	[28]
	AF205454	CA-63	[30]
	AF205453	CA-70	[30]
I B	AF205495	TX-68	[28]
	AF205491	TX-87	[28]
	AF205492	TX-89	[28]
II A	AF205472	BRA-68	[28]
	AF205460	FL-69A	[28]
	AF205459	FL-69B	[28]
II B	AF205499	TX-91	[28]
	AF205498	TX-83	[28]
II C	EF158062	FL-79	[29]
	AF205465	CA-89	[28]
	AF205466	TN-75	[30]
II D	AF205468	FL-62C	[28]
	AF205470	MEX-65	[28]
	AF205471	PAN-73A	[28]
II E	AF205508	MO-37	[28]
	AF205509	MO-33	[28]
II F	AF205513	GUA-69	[28]
III	AF205490	ARG-79	[30]
IV	AF205489	PAN-77A	[28]
	AF205488	PAN-U	[28]
	AF205476	PAN-73B	[28]
V A	AF205480	BRA- U	[28]
	AF205482	BRA-73B	[28]
	AF205481	BRA- ARG78	[28]
V B	AF205485	BRA-60	[19]
	AF205484	BRA-71	[19]
	AF205483	BRA-72	[19]
	EU906868	F72M022	[33]
VI	AF205487	PAN-83	[30]
VII	AY632544	ARG-66	[30]
	EF158068	ARG-67	[30]
VIII A	GU808548	BRA-84D	[19]
	GU808554	BRA-84F	[19]
	GU808556	BRA-84G	[19]
VIII B	GU808559	BRA-84J	[19]
	GU808546	BRA-84C	[19]
	GU808552	BRA-73F	[19]
WNV	HQ537483	Greece-10	[42]
JEV	AF148899	Austrália-98	[43]
DENV	EU448412	Taiwan-03	[44]

* Designation according to country or state (in the case of USA) and year of virus isolation. Abbreviations: CA, California; TX, Texas; NM, New Mexico; TN, Tennessee; FL, Florida; MD, Maryland; PAN, Panama; MEX, Mexico; GUA, Guatemala; ARG, Argentina; BRA, Brazil; WNV, West Nile Virus; JEV, Japanese Encephalitis Virus; and DENV, Dengue Virus.

### Virus isolation

A tissue fragment of the SLEV-positive brain was homogenized and clarified by centrifugation. The brain homogenate was inoculated on monolayers of the mosquito cell lineage C6/36 and cultures were monitored for cytopathic effect (CPE) daily. Viral stocks were collected for up to five days post-infection and re-inoculated (500 µL of supernatant on a fresh culture) two times, for a total of three passages. The viral titer from supernatant of the third passage was determined by focus immunodetection assay (FIA) as previously described [32]. We obtained SLEV stocks at 1×10^3^ focus forming units, or FFU, per mL of culture supernatant.

### Virus characterization *in vivo*

To characterize the isolated SLEV strain, 40 µL of SLEV stocks were inoculated in newborn mice by intracranial route (frontal left region of the brain). Homogenates of a pool of brain samples (10% in PBS) from the littermates were used for preparing the inoculum for the next passage. Infected newborn brains were collected at the onset of neurological disease or at day 7 post-infection, to produce new virus stocks or to process for histological analysis. Brain suspensions were passed seven times in newborn mouse brains and relevant experimental controls were maintained. Clinical alterations were assessed daily in newborns after each SLEV passage and representative pictures were taken.

### Histology

Tissue samples, including brain, kidney, liver, lung, heart, and fragments of the thoracic and pelvic limbs, were obtained from two newborn mice at each SLEV passage, immediately fixed in 4% buffered formaldehyde, processed and embedded in paraffin. Tissue sections (4 µm thick) were stained with hematoxylin and eosin (HE), and examined under light microscopy. Micrographs were taken using a Spot Insight color camera coupled to Olympus BX41 microscope.

### Immunofluorescence microscopy

C6/36 cells were harvested and seeded in 24-well plates with gelatin-coated coverslips, and incubated at 28°C for at least 3 hours. Cells were infected with a virus stock derived from the 7^th^ SLEV passage in newborn mice, at a multiplicity of infection (MOI) of 1, for 1 h. At 24 hours post-infection, cells were fixed, permeabilized and stained with a mouse anti-SLEV monoclonal antibody (MSI-7, clone 6B6C-1, MAB8744; Merck Millipore, USA) followed by Alexafluor 488-labelled secondary anti-mouse (Molecular Probes, Invitrogen, USA). Experiments were performed with control group with or without primary antibodies. Stained coverslips were mounted in Mowiol 4–88 (Polysciences, Inc., USA) and analyzed using an Olympus BX61WI microscope equipped with a FV300 confocal scanning unit. Images were analyzed with imageJ software.

## Results

### Identification of SLEV infection in a sick horse

From a total of 170 brain samples from horses with neurological disease received and analyzed by PCR at the Laboratório de Patologia Molecular at UFMG, one sample was identified as positive for SLEV. This brain sample was obtained in March 2009 (late summer) from a 12 years-old male horse of undefined breed, which died 72 hours after presenting neurological signs. Those neurological signs were described as incoordination, depression, and flaccid paralysis of the hind limbs. The horse came from a farm in Abaeté, countryside of Minas Gerais State, 207 kilometers from the capital, Belo Horizonte. In the same farm there were two additional horses that remained clinically healthy. Importantly, this SLEV-positive brain sample was negative for other pathogens commonly associated with encephalitis in horses, including Rabies virus, Equine Herpesvirus-1, Equine Herpesvirus-4, West Nile virus, Eastern equine encephalitis virus, Western equine encephalitis virus, Venezuelan encephalitis virus, and *Sarcocystis neurona*.

The SLEV amplicon originated by the RT-PCR reaction was sequenced and deposited in GenBank (Submission ID #1663732), referring to SLEV strain MG150.

**Figure 1:**
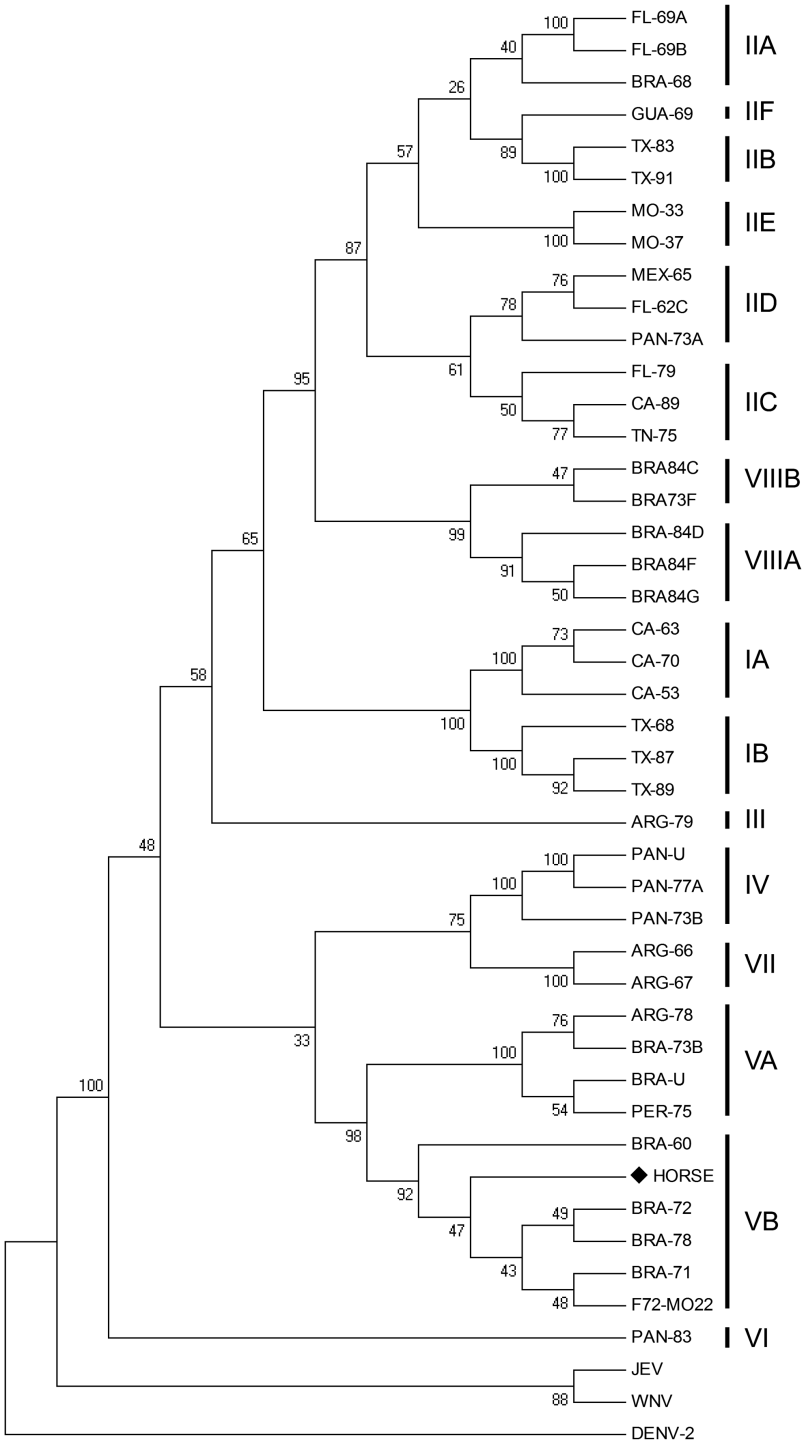
Analysis of the envelope (E) gene sequence of SLEV. Phylogenetic tree was constructed using partial E gene sequence by neighbor-joining (NJ) method (using p-distance) of 1,000 bootstrap replicates using Mega 4.0.2. Boostraps values above 60% are shown. The scale bar represents 5% nucleotide sequence divergence. WNV, JEV and DENV-1 were used as the out group.

Phylogenetic analysis of a 903 bp amplified sequence from partial Envelope (E) gene region [26] indicated that the isolate from the horse was within the cluster of the VB genotype (Figure 1). A higher degree of nucleotide identity (97–98%) was observed among ten SLEV strains from the Brazilian Amazon region, of the VB genotype (BRA-71, BRA-78, BRA-72, BRA-60, BRA-84B, BRA-74B, BRA68B, BRA-84A, BRA-74D, BRA-73E) by comparison with nucleotide sequences previously deposited in GenBank using BLAST algorithm (http://blast.ncbi.nlm.nih.gov/). Among the foreign isolates with a similar level of nucleotide identity (i.e. 98%), one (F72M022), also genotype VB originated in Florida from opossum in 2006 [33].

### Virus isolation and characterization

After confirmation of the virus identity by sequencing, we focused our efforts on isolating SLEV from the horse tissue. A brain fragment from the SLEV-positive horse was homogenized and inoculated in C6/36 mosquito cells, which is a cell lineage suitable for arbovirus propagation. All passages were tested for SLEV by RT-PCR, and were all positive beginning at the second passage. The virus was isolated after three passages, obtaining a SLEV stock of 1×10^3^ FFU/mL of supernatant.

**Figure 2:**
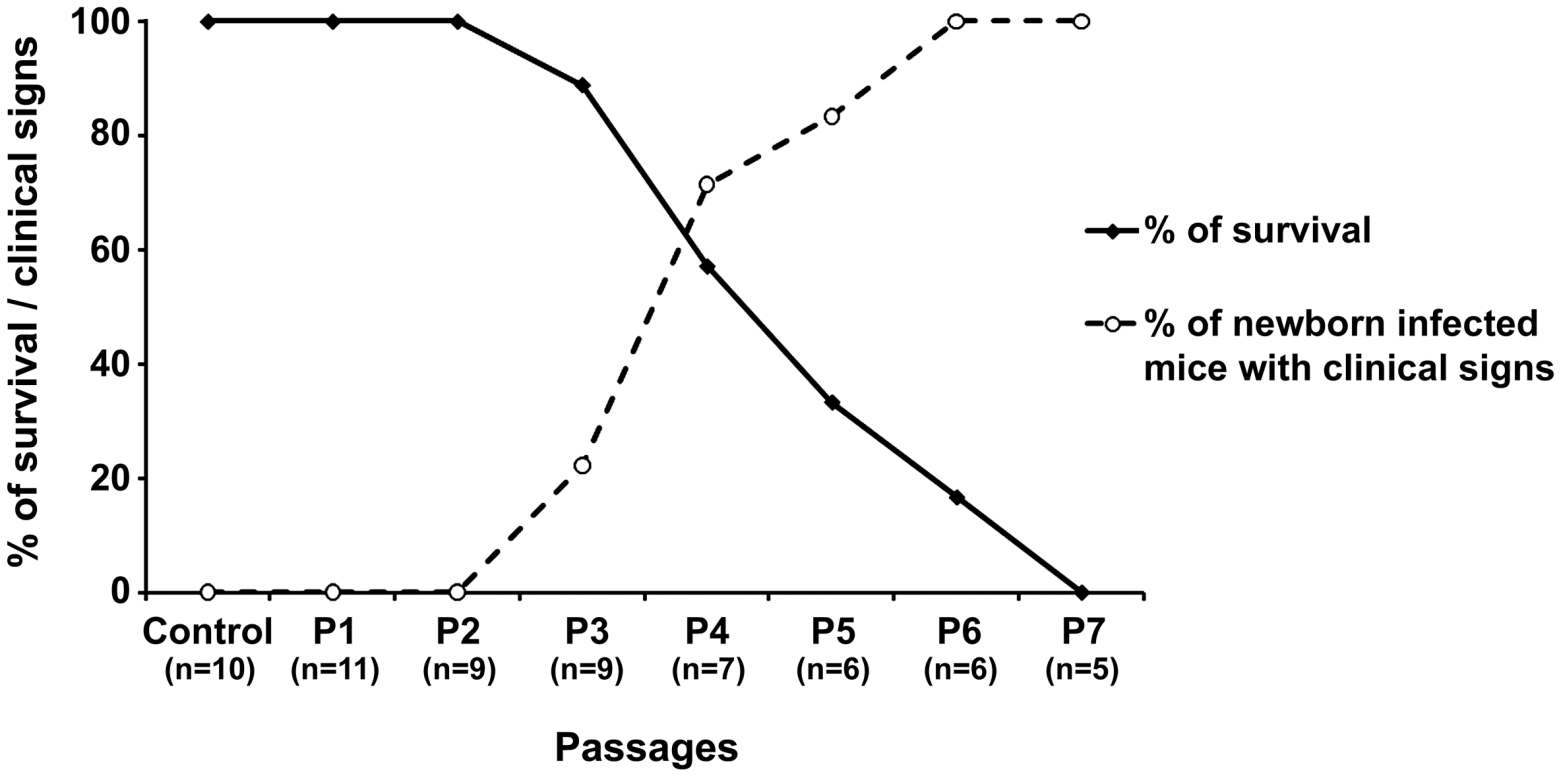
Survival rate and frequency of clinical signs in newborn mice inoculated with SLEV. Uninfected control mice (control), first (P1), second (P2), Third (P3), 4^th^ (P4), 5^th^ (P5), 6^th^ (P6), and 7^th^ (P7) passages are indicated in the X axis.

**Figure 3:**
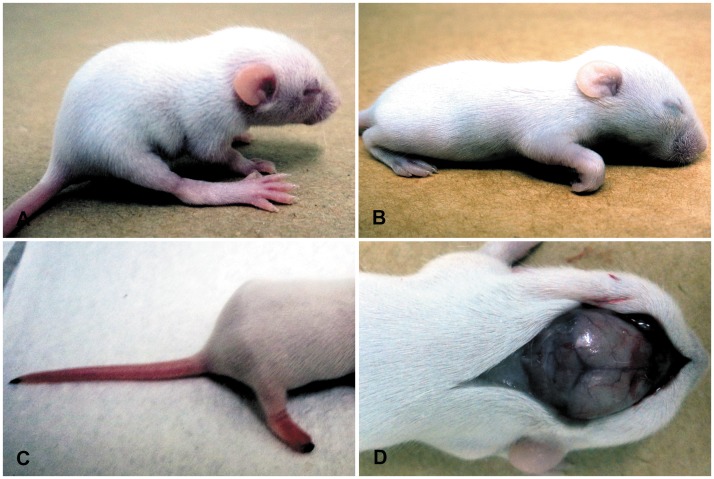
Newborn mice inoculated with SLEV presenting neurological and circulatory disorders at the 3^rd^ viral passage. (A) Bending of the spine (thoracic kyphosis), and limited mobility. (B) Front limb flexed characterizing loss of proprioception and apathy. (C) Necrosis of the distal extremity of the hind limbs and at the tip of the tail. (D) Hyperemia and extensive areas of hemorrhage in the meninges at the rostral part of the brain.

To characterize this newly isolated strain *in vivo*, we performed serial passages of SLEV by intracranial inoculation of newborn mice, which allowed us to gather some data on MG150 strain pathogenicity. Increase of clinical signs and circulatory changes were associated with increased mortality that reached 100% at the 7^th^ passage (Figure 2). At third passage, edema and necrosis at the distal extremity of the hind limbs and at the tip of the tail were observed in SLEV infected newborn mice (Figure 3). After the fourth passage SLEV infection was associated with behavioral changes ranging from excitability to apathy and neurological changes including tremors, loss of proprioception, and walking in circles, which were accompanied by the same circulatory changes as observed at the third passage (Figure 3). Importantly, neurological changes in the final SLEV passages were accompanied by hemorrhage in the CNS and peritoneum (Table 2). Neither mortality nor clinical signs were observed in uninfected control mice.

**Table 2:** Clinical signs and gross changes in newborn mice during experimental infection with SLEV.

Group^1^	Behavioral changes^2^	Neurological changes^3^	Necrosis^4^	Edema^5^	Hemorrhage^6^
Control	0/10 (0%)	0/10 (0%)	0/10 (0%)	0/10 (0%)	0/10 (0%)
P1	0/11 (0%)	0/11 (0%)	0/11 (0%)	0/11 (0%)	0/11 (0%)
P2	0/9 (0%)	0/9 (0%)	0/9 (0%)	0/9 (0%)	0/9 (0%)
P3	0/9 (0%)	0/9 (0%)	2/9 (22%)	2/9 (22%)	0/9 (0%)
P4	3/7 (43%)	4/7 (57%)	0/7 (0%)	5/7 (71%)	0/7 (0%)
P5	5/6 (83%)	3/6 (50%)	0/6 (0%)	5/6(83%)	5/6 (83%)
P6	6/6 (100%)	6/6 (100%)	0/6 (0%)	4/6 (67%)	6/6 (100%)
P7	5/5 (100%)	5/5 (100%)	0/6 (0%)	3/5 (60%)	5/5 (100%)

1 Negative control newborn mice, newborn mice from passages 1 to 7.

2 Kyphosis, hyperexcitability, lethargy.

3 Tremors, loss of proprioception, walking in circles.

4 Necrosis in posterior limbs and tip of tail.

5 Edema of subcutaneous and limbs.

6 Hemorrhages in CNS, abdominal cavity.

**Figure 4:**
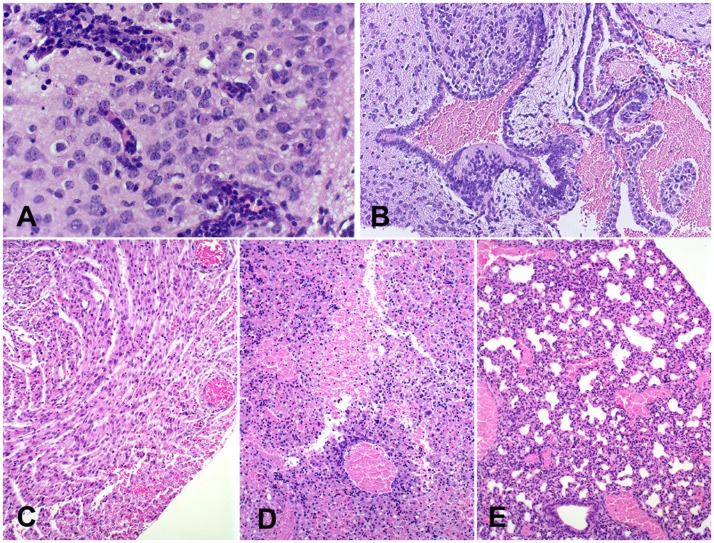
Central nervous system from newborn mice infected with SLEV. (A) Cerebral cortex from a newborn mouse at the 4^th^ viral passage with mild hyperemia and mild lympho-histiocytic perivascular cuffs, HE, 600×. (B) Choroid plexus from a newborn mouse at the 4^th^ viral passage, focally extensive hemorrhage in the ventricular cavity, HE, 200×. (C) Heart from a newborn at the 5^th^ viral passage, with moderate hyperemia and mild multifocal hemorrhage in the myocardium, HE, 200×. (D) Liver from a newborn at the 6^th^ viral passage, with severe hyperemia and focal hemorrhage, HE, 200×. (E) Lung from a newborn at the 7^th^ viral passage, with severe hyperemia, HE, 200×.

Histological analysis indicated hyperemia and discrete multifocal hemorrhage in CNS from all infected newborn mice at the 4^th^ passage. Multifocal mild lympho-hystiocytic inflammatory infiltrate in the leptomeninges and around blood vessels in the brain was observed in one newborn mouse at the 4^th^ passage (Figure 4A). Focal mild neuronal degeneration, and multifocal hemorrhage were also observed (Figure 4B). Circulatory changes such as hyperemia and multifocal hemorrhage were noticed in several organs, including brain, kidney, liver, lung, and heart at the 5^th^ passage (Figure 4C). Newborn mice at the 6^th^ passage also developed hyperemia in the kidney and lung, and multifocal hemorrhage in liver and brain (Figure 4D). Newborn mice at the 7^th^ passage had hyperemia in the kidney, liver, brain, and lung (Figure 4E). No histological changes were detected in uninfected control mice.

Together, these data indicates that SLEV can cause disease in newborn mice. Virus adaptation to the murine host increased after each passage, resulting in neurological and circulatory changes consistent with *Flavivirus* infection, providing further evidence that our isolated virus strain was indeed SLEV.

Inocula (i.e. CNS tissue homogenate pools) resulting from each passage in mice were submitted for RNA extraction for confirmation of viral detection by RT-PCR (data not shown). The same procedure was performed with organs that had gross changes. Pool of organs, including the liver, heart, kidney, and lung from the 4^th^ to the 7^th^ passage were analyzed. CNS and other organs were positive in all RT-PCR assays, confirming the presence of viral RNA in tissues.

### Detection of SLEV proteins in cell culture

To further confirm SLEV identity, we performed an immunofluorescence assay, using a commercial monoclonal antibody to detect SLEV proteins in cell culture. Monolayers of C6/36 mosquito cells were infected with the 7^th^ SLEV passage, fixed and stained 24 hours post-infection. Infected cells were positively stained with the anti-SLEV antibody, which was not observed in mock-infected cells, confirming the identity of this SLEV strain in an antibody-based test (data not shown).

## Discussion

The first isolation of SLEV from a horse that died due to a neurological disease in Brazil is a significant event. Virus isolation from horse brain tissue, together with molecular and immunofluorescence data, confirms that SLEV was the agent that caused disease and, ultimately, the horse death in this case. To our knowledge, this is the first observation that SLEV can cause disease in wild or domestic animals, which indicate that some aspects of SLEV viral cycle and its ability to cause disease need further studies. Furthermore, a model of newborn mice infection for characterization of SLEV was thoroughly described.

In terms of public health and epidemiology, the first identification and isolation of SLEV in the State of Minas Gerais adds to previous reports regarding SLEV detection in Brazil [2], [19], [21], [23], [24], and neighboring South American countries [34], [35], which strongly indicates that SLEV circulates in Brazil. Importantly, for SLEV epidemiological surveillance purposes, dengue is endemic and is an important health problem in Brazil. Antigenic similarity between SLEV, DENV and other flaviviruses, especially in terms of their envelope protein, generates cross-reactive antibodies that make serological detection of SLEV infections problematic, especially during the frequent dengue outbreaks [36]. Furthermore, SLEV infection can cause febrile illness and even hemorrhagic manifestations that are indistinguishable from mild and severe dengue fever cases, respectively [37]. In spite of these difficulties, molecular screening methods are available and could be employed to monitor SLEV circulation, allowing for preparedness in case of virus re-emergence or SLEV encephalitis outbreak, which has taken place in Argentina [38] and several times in the United States [39]. Considering that the SLEV strain isolated in this study came from a horse with neurological signs, and that the virus was able to induce systemic and neurological sings in mice, the virulence of the circulating strains should be evaluated. A serological survey involving five Brazilian states, including Minas Gerais, resulted in a prevalence of 36% with a total of 753 horses sampled [40].

Despite the existence of eight lineages and fifteen subtypes of SLEV, namely IA, IB, IIA, IIB, IIC, IID, IIG, III, IV, VA, VB, VI, VII, VIIIA, and VIIIB, phylogenetic studies based on the E gene indicate that genotypes I and II are found predominantly in North America, whereas genotypes III to VIII have been isolated in South and Central Americas [19]. Brazilian SLEV isolates have been classified within the genotypes II, III, V (A and B), and VIII (A and B). Genotypes V and VIII are predominately Brazilian Amazon region, whereas genotypes II and III have been isolated in the State of São Paulo (Southeast Region). In this study, the isolate from a horse had a higher degree of identity (97–98%) with the VB genotype, suggesting that this sample was likely originated from the Brazilian Amazon Region. The circulation of SLEV from the Amazon Region in the Southeast Region of Brazil suggests a possible involvement of migratory birds in disseminating the virus, since SLEV has been detected in 49 species of wild birds in Brazil, many of which are migratory [19], [21]. SLEV strains genotype VB were isolated since 1960s from wild birds and mosquitoes or sentinel animals at a surveillance site for arboviroses in a forested area of Pará state [19]. Migratory birds may have also been related to the periodic introduction of South American SLEV genotype V in Florida (USA) in 2006, originated possibly from Brazil, Mexico, or Panama [33].

In our efforts to characterize SLEV strain MG150 *in vivo*, to study its pathogenicity, we noticed the virus progressively adapted to serial passagens in newborn mice. The mouse has been previously used as a model for assessing SLEV virulence [41]. Disease presented by newborn mice infected with the last SLEV passages had some similarities to human disease, such as the development of hemorrhagic manifestations [19], mortality, and neurological changes [12], [14]. Importantly, lesions in organs such as the liver and heart are likely to reflect a systemic circulatory change rather than any specific viral tropism for these organs.
